# Retrospective immunohistochemical analysis of human cytomegalovirus infection in the placenta and its association with fetal growth restriction

**DOI:** 10.20407/fmj.2022-001

**Published:** 2022-07-22

**Authors:** Yusuke Funato, Yuki Higashimoto, Yoshiki Kawamura, Yoshiko Sakabe, Minori Iwakura, Masaru Ihira, Kazuya Shiogama, Masafumi Miyata, Haruki Nishizawa, Takao Sekiya, Takuma Fujii, Isao Kosugi, Tetsushi Yoshikawa

**Affiliations:** 1 Department of Pediatrics, Fujita Health University, School of Medicine, Toyoake, Aichi, Japan; 2 Faculty of Medical Technology, Fujita Health University, School of Medical Sciences, Toyoake, Aichi, Japan; 3 Department of Obstetrics and Gynecology, Fujita Health University, School of Medicine, Toyoake, Aichi, Japan; 4 Faculty of Clinical Engineering, Fujita Health University, School of Medical Sciences, Toyoake, Aichi, Japan; 5 Faculty of Pathology, Fujita Health University, School of Medical Sciences, Toyoake, Aichi, Japan; 6 Department of Regenerative and Infectious Pathology, Hamamatsu University, School of Medicine, Hamamatsu, Shizuoka, Japan

**Keywords:** Human cytomegalovirus, Congenital infection, Growth restriction, Immunohistochemistry, Placenta

## Abstract

**Objectives:**

Fetal human cytomegalovirus (HCMV) infection might be involved in fetal growth restriction (FGR). Maternal serostatus and the prevalence of congenital HCMV infection are affected by various factors, such as socioeconomic status and ethnicity. Therefore, the prevalence of congenital HCMV-related FGR should be examined in each region.

**Methods:**

Seventy-eight cases of FGR with delivery between January 2012 and January 2017 at Fujita Health University Hospital were studied. Twenty-one non-FGR cases were also included as a control group. Placental sections obtained from the FGR and control cases were immunostained with two primary antibodies for detecting immediate early antigens.

**Results:**

Nineteen placental samples from FGR cases with another etiology were excluded. Finally, 59 placental samples from FGR cases of unknown etiology were included in the pathological analysis. Four of 59 (6.8%) placental samples were positive for HCMV antigen. All four positive cases were stained with the M0854 antibody, and there were no positive case with the MAB810R antibody. Neither maternal nor infantile clinical features were different between the HCMV-positive and -negative FGR cases. A pathological examination showed a hematoma in three of four cases and infarction in two of four cases.

**Conclusions:**

HCMV antigen was detected in 6.8% of placental samples obtained from FGR cases without an obvious etiology. No remarkable maternal or neonatal clinical features discriminated HCMV-related FGR from FGR due to other causes. Vasculitis and inflammation might play important roles in the pathogenesis of HCMV-related FGR.

## Introduction

Human cytomegalovirus (HCMV) is one of the most common pathogens causing congenital infections worldwide. Symptomatic infection can cause microcephaly, neurological deficiencies, hearing loss, chorioretinitis, and fetal growth restriction (FGR).^1^ Primary maternal HCMV infection in the first trimester of pregnancy is associated with a 40%–50% risk of viral transmission to the fetus, which results in fetal damage.^2,3^ Recurrent HCMV infection is more common than primary infection, but the risk of viral transmission is lower. Additionally, HCMV infection can cause milder clinical manifestations such as hearing impairment.^4^ To prevent hearing impairment due to congenital HCMV infection, the cost-effectiveness of newborn hearing screening testing has been investigated.^5^ Activities to inform pregnant women about congenital HCMV infection are also important to reduce the risk of maternal infection. Contact precautionary measures, such as hand hygiene during pregnancy, especially in the first trimester, are necessary to reduce the number of patients with congenital HCMV infection.

Various factors, including poor maternal nutrition, chronic hypertension, diabetes, genetic abnormalities, smoking, preeclampsia, and fetal infection (e.g., HCMV), are involved in FGR. FGR occurring with maternal HCMV infection might be due to direct HCMV infection of the fetus^6,7^ or indirect effects such as cytokine upregulation.^8^ FGR is also caused by placental HCMV infection without fetal infection.^8–10^ Because maternal serostatus and the prevalence of congenital HCMV infection are affected by various factors, such as socioeconomic status, race, and ethnicity,^11,12^ the prevalence of congenital HCMV-related FGR should be examined in each region. Therefore, this study aimed to detect HCMV infection in placentas obtained from FGR cases using immunohistochemical analysis in Aichi Prefecture, which is located in the central region of Honshu Island in Japan.

## Methods

### Subjects and sampling

This study was performed in accordance with appropriate clinical and experimental ethical guidelines. The study was approved by the institutional review board of our university (HM18-319). Consent for patients to participate in this study was obtained through an opt-out method because stored paraffin-embedded placental samples were retrospectively used for pathological analysis in this study.

Seventy-eight cases of FGR with delivery between January 2012 and January 2017 at Fujita Health University Hospital were included in this study. FGR was defined as an estimated fetal body weight below –1.5 standard deviations (SDs) on a predelivery ultrasound evaluation.^13^ Cases with an obvious etiology for FGR, including chromosomal aberrations, multiple conceptions, or hydatidiform mole, were excluded from the pathological analysis. Additionally, 21 cases without FGR were also investigated as a control group. The characteristics of the 21 control cases were as follows: 16 involved threatened preterm labor, 2 involved placenta previa, 4 involved preeclampsia, and 3 involved premature rupture of the membranes. Some cases had two concurrent complications. The median maternal age of the control cases was 32 years (27–35 years), and the mean gestational age of the neonates was 34.2±0.8 weeks. The median birth weight percentile, birth height percentile, and birth head circumference percentile of the neonates were 45.4 (33.9–61.3), 60.4 (50.7–87.7), and 60.8 (44.3–80.2), respectively. The median placental weight was 450 (420–530) g.

Clinical characteristics and laboratory findings of the pregnant women and their newborns were obtained from medical records. The retrospectively collected information included maternal age, gestational age, preeclampsia status, neonatal sex, birth weight, birth height, head circumference at birth, Apgar score, Kaup index, placental weight, and pathological findings of the placenta, such as chorioamnionitis, funisitis, infarction, and hematoma.

### Immunohistochemistry

Tissue specimens obtained from two parts of each placenta were fixed in formalin and embedded in paraffin. Immunohistochemistry was performed as follows. Serial 5-μm-thick tissue sections were deparaffinized with xylene and rehydrated in graded ethanol. Endogenous peroxidase was quenched with 3% hydrogen peroxide solution. Antigen retrieval was then performed. Heat-induced epitope retrieval was performed using a pressure pan (Delicio 6L; T-FAL, Ecully, France) for 10 minutes. The soak solution consisted of 1 mM ethylenediaminetetraacetic acid solution at pH 8.0. Placental sections were immunostained with two primary antibodies (clone CCH2+DDG9 [M0854; Dako, Glostrup, Denmark] diluted 1/200 and clone 8B1.2 [MAB 810R; Millipore, Burlington, USA] diluted 1/800). Sections were incubated with the two primary antibodies overnight. The slides were incubated with anti-mouse and anti-rabbit universal immunoperoxidase polymer (Histofine Simple Stain MAX-PO; Nichirei Biosciences, Tokyo, Japan), rinsed, and incubated for 30 minutes. The reaction products were visualized with diaminobenzidine tetrahydrochloride (Agilent Technologies, Santa Clara, CA, USA) and counterstained with hematoxylin. Phosphate-buffered saline was used as the primary antibody for a negative control. Liver sections obtained from HCMV-infected patients were used as a positive control.

### Statistical analysis

Statistical comparisons between FGR cases with and without HCMV infection were performed with the Mann–Whitney U test in EZR <https://www.jichi.ac.jp/saitama-sct/SaitamaHP.files/statmed.html>. All reported *p* values are two-sided. Statistical significance was defined as *p*<0.05.

## Results

**Figure 1:**
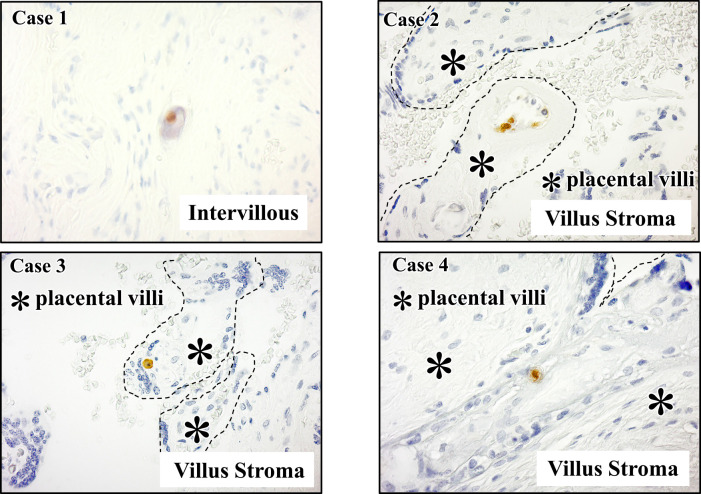
Placental human cytomegalovirus (HCMV) immunohistochemical staining. Viral antigen was detected in the villous stroma (fetal part) in Cases 2, 3, and 4, and in the intervillous space (maternal part) in Case 1. Dotted lines indicate the surface of the placental villi.

Nineteen cases with an obvious etiology for FGR were excluded from the pathological analysis as follows: 7 cases of chromosomal abnormalities, 11 cases of multiple conception, and 1 case of hydatidiform mole. Ultimately, placental tissue obtained from 59 FGR cases was included in the pathological analysis. Four of 59 (6.8%) placental samples were positive for HCMV antigen. All four positive cases were stained with the M0854 antibody. No cases had positive staining with the MAB 810R antibody. Viral antigen was detected in the villous stroma (fetal part) in three cases and in the villous lumen (maternal part) in one case (Figure 1). No HCMV antigens were detected in placental samples obtained from the 21 control cases without FGR.

Among the FGR cases, maternal clinical features were compared between HCMV-positive and HCMV-negative cases (Table 1). There were no significant differences in maternal age, gestational age, preeclampsia status, HCMV serostatus, placental weight, or pathological findings of chorioamnionitis, infarction, or hematoma between the two groups. Clinical features of the neonates were also compared between HCMV-positive and HCMV-negative cases (Table 2). There were no significant differences in sex, birth weight, birth height, head circumference at birth, the Apgar score, or the Kaup index between the two groups (Table 2).

The clinical features and pathological findings of the four HCMV-positive cases are shown in Table 3. Three of four cases were small for gestational age (SGA); two of the three cases had an asymmetrical SGA status (Cases 2, 3, and 4). HCMV antigen was detected in the intervillous space in Case 1 and in the villous stroma in Cases 2, 3, and 4. Viral antigen was localized to the cell nucleus in Cases 1, 2, and 4, and the cytoplasm in Case 3. A hematoma was observed in Cases 1, 2, and 3. Infarction was observed in Cases 2 and 4. The final diagnosis was placental abruption for Case 1 and hypertensive disorder of pregnancy for Cases 2 and 4.

## Discussion

In this study, the prevalence of HCMV infection in placentas obtained from pregnant women that was associated with FGR was 6.8%, which is lower than that (71.4%) found in previous studies conducted in the United States.^10^ In a previous study,^10^ five of seven cases with intrauterine growth retardation were considered to have either recurrent or primary HCMV infection on the basis of serological analysis, which is more frequent than that usually reported (32%–50%).^14^ In a previous prospective Japanese study,^8^ 36 of 48 (75.0%) cases were seropositive for HCMV. Additionally, 10 of 48 (20.8%) cases were considered to have possible HCMV infection because HCMV immunoglobulin (Ig) M antibody was positive or there was low HCMV IgG antibody avidity. Because we performed a retrospective study, serological data were available for only 37 of 59 FGR cases, which is a limitation of this study. Twenty-two of 37 (59.5%) cases were HCMV-seropositive, and 7 of 37 (18.9%) cases were positive for HCMV IgM antibodies. This finding suggests a similar HCMV serostatus to that in a previous Japanese prospective study.^8^ Another retrospective study conducted in Japan also showed that 6 of 319 (1.8%) FGR cases were infected with HCMV, which suggested a low incidence of HCMV-related FGR.^15^ In this previous study, congenital infection was defined by maternal serology and urine CMV polymerase chain reaction in neonates. The specificity of the HCMV IgM assay is not sufficient for a precise diagnosis of maternal HCMV infection because IgM can persist for a long time in some patients.^16^ Additionally, the reliability of the IgG avidity assay for distinguishing between recent and past infections remains unclear.^17–19^ Because of the low frequency of HCMV infection in FGR cases, in addition to reliable serological examinations, pathological analysis of placental tissue should be performed in more cases to determine the precise clinical features of HCMV-related FGR.

In this study, placental samples obtained from FGR cases and control cases without FGR were examined to evaluate the association between HCMV infection and FGR. Twenty-one control cases were analyzed in this study, but HCMV antigen was not detected in control placental samples. However, there were no significant differences in HCMV positivity in placental samples between FGR cases and controls in this study. The reasons for this finding are the low positivity for HCMV antigen in FGR cases and the small number of control cases. Therefore, large numbers of FGR and control cases should be examined using pathological analysis to confirm the positive correlation between maternal HCMV infection and FGR.

Determining the clinical characteristics for distinguishing between HCMV-related FGR and FGR of other etiologies is important from a clinical standpoint, but this study did not show any characteristic clinical features of HCMV-related FGR. A previous Japanese study also showed no significant differences in maternal clinical features, such as age, body mass index, and parity, between HCMV antigen-positive and HCMV antigen-negative cases.^8^ However, the prevalence of preeclampsia was higher in the HCMV-positive cases than in the HCMV antigen-negative cases. Comparison of fetal ultrasound sonographic findings of the two groups showed that the ΔSD of estimated fetal body weight, abdominal circumference, and femur length was significantly lower in the HCMV-positive cases than in the HCMV-negative cases.^8^ Although we compared neonatal findings at the time of delivery, no significant differences were found between the two groups. Further analysis of more cases is required to determine the clinical characteristics of HCMV-related FGR.

In this study, only M0854 antibody detected HCMV antigen in the four FGR cases. MAB 810R antibody did not detect any HCMV antigen. Although both antibodies have been reported to detect immediate early (IE) antigens, the MAB 8101R antibody reacts with IE1 and IE2 because it reacts with the protein encoded by exon 2, which is shared by IE1 and IE2.^20^ According to the manufacturer’s data sheet, the monoclonal antibody DDG9 component of M0854 detects only the 76-kD IE1 <http://webzis.biopticka.cz/Protilatky/store/CMV.pdf>. Therefore, the antibody reacts to the epitope encoded by exon 4. Furthermore, a small amount of IE1 exon 4 protein expression is required for viral genome maintenance during latency.^21^ The HCMV antigen detected in this study might be part of the small IE1 exon 4 protein, but not the entire IE1 or IE2 protein. Therefore, this possibility might reflect persistent or latent HCMV infection instead of lytic infection with viral replication in the antigen-positive cells. This concept is supported by the lack of typical morphological changes, such as cytomegalic inclusion bodies, observed in HCMV antigen-positive placental samples.

In addition to immunohistochemical analysis, gene expression analysis of the three classes of HCMV genes is required to determine the precise viral replication cycles in placental tissues. This information is important for clarifying the mechanisms of HCMV-related FGR. Many studies on the regulation of HCMV gene transcription have shown that three classes of genes are tightly regulated for virus replication.^22–27^ In addition to active and latent infection, the concept of a dynamic latent state has been proposed, which is characterized by low levels of viral gene expression in the absence of viral progeny production.^28^ Because only the IE antigen was detected in placental samples in this study, dynamic latent infection might have occurred.

Understanding how a small number of HCMV infections without full viral replication can cause FGR could be important. The bystander effect of HCMV infection through the host–immune response may play an important role in causing FGR. HCMV antigens can induce an innate immune response via a Toll-like receptor 2-dependent mechanism and cause placental inflammation without direct infection.^6,7^ Inflammation can cause large fibrinoids with many avascular or edematous villi, as seen in our cases, which could reduce perfusion and transport of substances across the placenta.^10^ Pereira et al. used the same antibody cocktails as those used in our study and found viral antigens in the smooth muscle cells of arteries and veins in floating villi and the chorion.^10^ Therefore, vasculitis and inflammation might play important roles in causing HCMV-related FGR. More in-depth analysis is required to determine the precise mechanism of HCMV-related FGR without active viral infection.

Because this was a retrospective analysis of stored placental tissues, no virological analysis was conducted in the neonates. Determining if the placenta alone or in combination with fetal infection is required to cause HCMV-related FGR is important. Therefore, a prospective study for analyzing the placenta and neonate together is currently underway.

## Figures and Tables

**Table1:** Maternal characteristics and pathological findings of the placenta

	Placental CMV (–) Group (n=55)	Placental CMV (+) Group (n=4)	*P* value
Maternal age (y)	32 (29.0–35.0)	34 (29.5–38.5)	0.397
Gestational age (week)	35 (33.0–37.0)	34 (32.5–35.5)	0.637
Preeclampsia (%)	21 (38.1)	2 (50)	0.661
CMV-IgM positive (%)	7 (21.2)	1 (25)	1.0
CMV-IgG positive (%)	20 (60.6)	2 (50)	1.0
Placental weight (g)	350 (282.5–420)	380 (320–411.25)	0.818
Chorioamnionitis Funisitis (%)	8 (14.5)	0 (0)	0.412
Infarction Hematoma (%)	38 (69.1)	4 (100)	0.456

Date are expressed as median (IQR) or n (%)

**Table2:** Characteristics of neonates

	Placental CMV (–) Group (n=55)	Placental CMV (+) Group (n=4)	*P* value
Male sex	29 (52.7)	2 (50)	0.916
Birth weight percentile	0.87 (0.13–2.98)	0.89 (0.20–8.35)	0.940
Birth height percentile	1.62 (0.13–2.98)	2.88 (1.62–7.91)	0.775
Birth head circumstance percentile	9.74 (3.14–20.1)	22.4 (0.36–52.3)	0.861
Apgar score 1 min ≥7 (%)	36 (65.5)	4 (100)	0.533
Apgar score 5 min ≥7 (%)	48 (87.3)	4 (100)	0.708
Kaup index (kg/m^2^)	9.46 (8.55–10.8)	9.75 (9.03–10.2)	0.880

Date are expressed as median (IQR) or n (%)

**Table3:** Patients’ background and pathological findings of the placenta in HCMV-positive cases of FGR

Cases	Maternal age	FGR (SD)	HCMV localization	Pathologic findings	Diagnosis
1	30	–2.0	Intervillous	Hematoma	Placental abruption
2	38	–2.6	Villus stroma	Infarction Hematoma	HDP
3	28	–2.9	Villus stroma	Hematoma	—
4	40	–2.7	Villus stroma	Infarction	HDP, NRFS

HDP, hypertensive disorder of pregnancy; NRFS, Non reassuring fetal status
