# Supplementary material for: Retrospective immunohistochemical analysis of human cytomegalovirus infection in the placenta and its association with fetal growth restriction
Source: Fujita Med J. 2022 Jul 22;9(2):90–4. doi: 10.20407/fmj.2022-001 (PMC10206905; doi:10.20407/fmj.2022-001)

Supplementary material. Placental immunohistochemical staining from an HCMV antigen-negative mother.

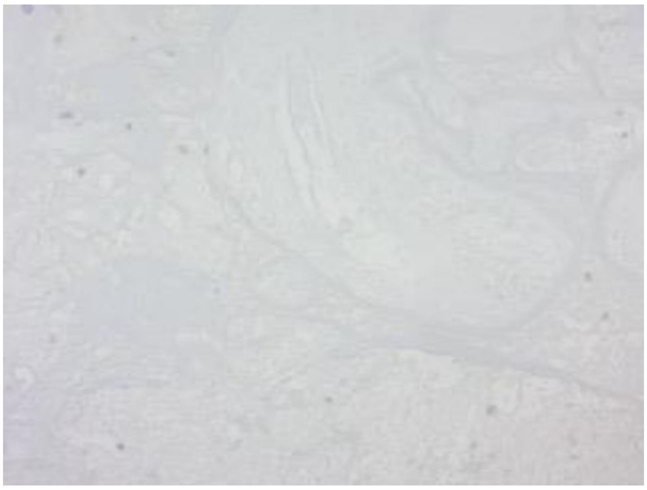

Supplement: Supplementary file 1 — Supplementary Figure [file fmj-9-090-s001.pdf]
